# Efficacy of Immunohistochemistry for SDHB in the Screening of Hereditary Pheochromocytoma–Paraganglioma

**DOI:** 10.3390/biology10070677

**Published:** 2021-07-17

**Authors:** Hye-Ryeon Choi, Ja-Seung Koo, Cho-Rok Lee, Jan-Dee Lee, Sang-Wook Kang, Young-Seok Jo, Woong-Youn Chung

**Affiliations:** 1 Department of Surgery, Eulji Medical Center, Eulji University School of Medicine, Seoul 01830, Korea; hrchoi@eulji.ac.kr; 2 Department of Pathology, Yonsei University College of Medicine, Seoul 03722, Korea; 3 Department of Surgery, Yonsei University College of Medicine, Seoul 03722, Korea; crlee@yuhs.ac (C.-R.L.); jandee@yuhs.ac (J.-D.L.); woungyounc@yuhs.ac (W.-Y.C.); 4 Department of Internal Medicine, Yonsei University College of Medicine, Seoul 03722, Korea; joys@yuhs.ac

**Keywords:** pheochromocytoma, sympathetic paraganglioma, genetic screening, immunohistochemistry, SDHB

## Abstract

**Simple Summary:**

The majority of hereditary pheochromocytoma and paraganglioma (PPGL) is caused by germline mutations in the *SDHB, SDHC,* or *SDHD* genes, which encode three (SDHB, SDHC, SDHD) of five subunits of succinate dehydrogenase (SDH) enzyme (SDHA, SDHDB, SDHC, SDHD, SDHAF2). The inactivation of any of the *SDHx* genes leads to the proteolysis of SDH. This can be detected with negative immunohistochemistry (IHC) for SDHB. However, there is no consensus of the interpretation of “weakly positive” staining. Hence, we carried out a retrospective single centre study comprising 58 patients to evaluate the efficacy of using SDHB IHC for detecting *SDHx* mutation in PPGL cases. Our analyses revealed that SDHB-negative IHC is a cost-effective and reliable method to predict *SDHx* mutations in the Korean population. Nonetheless, in the case of weakly positive staining, an additional gene study should be considered.

**Abstract:**

The most common genetic backgrounds of hereditary paraganglioma and pheochromocytoma (PPGL) are *SDHx* germline mutations. Given the fact that the immunohistochemistry (IHC) result for SDHB is always negative regardless of the type of *SDHx* mutation, we aimed to evaluate the efficacy of using SDHB IHC for screening *SDHx* mutations in PPGL cases. In total, 52 patients who underwent surgery for PPGL treatment between 2006 and 2020 and underwent genetic analysis at diagnosis were included. Tissue microarrays (TMAs) were constructed with PPGL tissues and IHC for SDHB was performed on TMA sections. All 10 patients with SDHB-negative IHC contained *SDHB* or *SDHD* mutations. The genetic test results of patients with SDHB-weakly positive IHC varied (one *SDHB*, two *RET*, one *VHL*, and three unknown gene mutations). There were no *SDHx* mutations in the SDHB-positive IHC group. Six patients with weakly positive SDHB IHC with primarily unknown genetic status were re-called and underwent next-generation sequencing. None of them had *SDHx* mutations. In conclusion, SDHB-negative IHC is a cost-effective and reliable method to predict *SDHx* mutations. However, in the case of weakly positive SDHB staining, an additional gene study should be considered.

## 1. Introduction

Pheochromocytomas and sympathetic paragangliomas (PPGLs) are rare catecholamine-secreting tumours arising from chromaffin tissue of the adrenal medulla or extra-adrenal sympathetic paraganglia [1]. The classical “rule of 10”, which states that 10% of these tumours are associated with a familial syndrome, has been invalidated recently. Rather, PPGL shows the highest degree of heritability in human neoplasms, up to 40% [2]. Although a sporadic form exists, the vast majority of PPGLs occur as a manifestation of inherited tumour syndromes such as multiple endocrine neoplasia type 2 (MEN2; *RET* gene), von Hippel–Lindau (VHL) disease (*VHL* gene), neurofibromatosis type 1 (NF1; *NF1* gene), and hereditary PPGL [3,4,5,6]. Among these, hereditary PPGL is the most common condition and is mostly caused by germline mutations in the *SDHB*, *SDHC*, or *SDHD* genes [7,8]. The rich genetic background of these tumours, which is even found in 12% of apparently sporadic PPGLs [9], has led to recommendations that every patient with PPGL should be considered for genetic testing [10]. Gene study allows the identification of other manifestations of hereditary tumour syndromes, verifying the mutations associated with a high risk of metastasis such as *SDHB* mutation, and the screening of at-risk probands [10].

The most common germline mutations are *SDHB* and *SDHD* genes, which account for 6–9% of sporadic PPGL and more than 80% of familial aggregations of PPGL [10,11]. PPGLs related to mutations of *SDHB* and *SDHD* genes usually occur early in life and are often multifocal [12]. Strong association between metastasis and *SDHB* mutation was also reported [7,13]. The penetrance of *SDHB* and *SDHD* germline mutations is known to be approximately 50% in patients in their 30s [6]. This incomplete penetrance, along with maternal imprinting (*SDHD*), the phenotypic heterogeneity of the disease, lack of family information, overlap in age distribution between hereditary and sporadic cases, de novo mutations, and insufficient awareness of clinicians, can lead to the underdiagnosis of hereditary PPGL [8].

Nowadays, next-generation sequencing (NGS) panels are widely used to evaluate germline mutation status. Although the introduction of NGS greatly reduced the cost and time of genetic testing compared to those of conventional Sanger sequencing [14], it is still meaningful to find a cost-effective screening tool that can detect *SDHx* mutation, which accounts for the largest portion of hereditary PPGL.

Succinate dehydrogenase (SDH) is a protein complex that plays an important role in the electron transport chain and the Krebs cycle [15]. The SDH complex is composed of two anchoring subunits (SDHC and SDHD) and two catalytic subunits (SDHA and SDHB). Each subunit is transcribed from *SDHC*, *SDHD*, *SDHA*, and *SDHB* genes, respectively. Remarkably, immunohistochemistry (IHC) for SDHB becomes negative if any component of the SDH complex is lost due to mutations of *SDHx* genes [16]. Therefore, efforts to validate IHC for SDHB as a cost-effective screening tool for hereditary PPGL have been made. Since van Nederveen et al. and Gill et al. reported the high sensitivity and specificity of negative IHC for SDHB to diagnose hereditary PPGL in the late 2000s [8,17], several similar studies have been published [17,18,19,20]. The fourth edition of the World Health Organization’s (WHO) classification of endocrine tumours mentioned negative IHC for SDHB as an important marker for this group of tumours [21].

However, the interpretation of “weakly positive” staining of SDHB is still in debate. In this first study in South Korea, we conducted IHC for SDHB in 52 genetically analysed patients with PPGL who underwent surgery at a large tertiary referral centre. Additional NGS for patients showing weakly positive SDHB IHC with primarily unknown genetic status was performed to evaluate the clinical meaning of weakly positive staining.

## 2. Materials and Methods

Formalin-fixed and paraffin-embedded (FFPE) tumour tissues of PPGL from 230 patients who underwent surgery at the Thyroid and Endocrine Surgery Centre of Yonsei Severance Hospital (Seoul, Korea) between 2006 and 2020 were available. Among those subjects, 52 patients who underwent genetic analysis at diagnosis either through NGS or direct sequencing of *RET*, *NF1*, *VHL*, and *SDHx* genes were included. Thirty five patients received NGS for germline mutation analysis and 17 received direct sequencing for RET, VHL and NF1. All patients who underwent direct sequencing test had a family history of pheochromocytoma and other clinical manifestations of MEN type 2 or neurofibroma. On the other hand, patients who underwent NGS were apparently sporadic. For additional analysis of weakly positive staining, 6 genetically untested patients with weakly positive SDHB IHC had been recalled and undergone NGS. All of these patients showed sporadic phenotype at diagnosis.

Medical records were retrospectively reviewed. Pheochromocytoma of the adrenal gland scaled score (PASS) was used to risk stratification. PASS consists of 12 histomorphological parameters. Tumours with PASS ≥ 4 are considered at high risk of metastasis.

### 2.1. Tissue Microarray (TMA) Construction

Haematoxylin and eosin (H&E)-stained slides were reviewed and the slide with the most representative tumour area was selected. Then, the matching FFPE blocks were collected. In the collected FFPE blocks, representative tumour areas were identified by contrasting with H&E-stained glass slides, and 3-mm cores were obtained and transferred to empty paraffin blocks to construct TMA blocks. The TMAs were sectioned in 4-µm slices, mounted on glass slides, and stained with H&E.

### 2.2. IHC of SDHB

IHC was performed on 4-μm-thick sections from TMA blocks. IHC was performed according to the manufacturer’s protocols. Briefly, SDHB staining was performed on an automated Ventana BenchmarkXT slide stainer (Ventana, Tucson, AZ, USA), using primary antibodies against *SDHB* (Abcam, clone 21A11, 1:50). Ultra VIEW Universal DAB Detection Kit (Ventana/Roche; Tucson, AZ, USA) was used in accordance with the manufacturer’s instructions. Human adrenal gland tissues were used as positive controls. In the tumour, the normal stromal cells or endothelial cells or lymphocytes served as an internal positive control for each sample. The primary antibody was omitted for negative control.

### 2.3. IHC Interpretation

**Figure 1:**
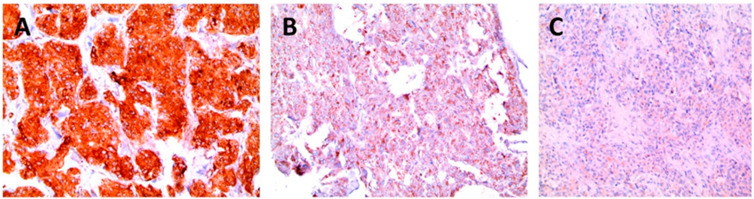
Positive (**A**), weakly positive (**B**), and negative (**C**) stains for SDHB.

All immunohistochemically stained slides were interpreted by one experienced endocrine pathologist. The pathologist interpreted the IHC slide without any knowledge of the clinical, pathologic, and molecular data. The interpretation criteria used in this study followed those reported in a previous study [17]. Briefly, cases with definite granular cytoplasmic staining of tumour cells (mitochondrial pattern) were considered positive. Cases with weak cytoplasmic expression with no granular cytoplasmic staining were classified as “weakly positive”. As in previous studies of Gill et al., the diffuse quality of the cytoplasmic staining of the tumour cells was identified in these cases, which was contrasted with the strong granular staining of internal controls [17]. Cases with focal but definite granular cytoplasmic staining of tumour cells were also considered positive. Figure 1 shows the positive, weakly positive, and negative stains for SDHB. The distinction between positive and weakly positive staining was based on the intensity of staining, not the proportion of staining. All “weakly positive” slides were reviewed again to confirm the interpretation.

### 2.4. Genetic Analysis

Mutation analyses for 52 patients were performed previously. DNA was retrieved from peripheral blood and isolated using standard procedures. Mutation analyses for RET, NF1, VHL, and SDHB genes were performed with either commercial sequencing of PCR products or with endocrine NGS panel of our centre.

#### 2.4.1. Sanger Sequencing

PCR amplification was performed using the in-house primers for RET, NF1, VHL, and SDHB sequencing, which were designed to amplify exon 10, 11, and 13–16 of RET, all exons of NF1, coding exons 1, 2, and 3 of VHL, and exon 1–8 of SDHB, respectively. After PCR-amplified reactions, the PCR products were treated with exonuclease I and shrimp alkaline phosphatase (USB Corp., Cleveland, OH, USA). Direct sequencing was performed using a 3730 DNA Analyzer with the BigDye Terminator v3.1 Cycle Sequencing Kit (Applied Biosystems, Foster City, CA, USA). Enzyme reactions and cycle reactions were carried out according to the manufacturers’ instructions. The results were aligned against reference sequences (NM_020630.4 for RET, NM_000267.3 for NF1, NM_000551.3 for VHL, and NM_0030002 for SDHB) using the Sequencher 5.3 software (Gene Codes Corp., Ann Arbor, MI, USA). Identified variants were annotated according to nomenclature recommendations of the Human Genome Variation Society (HGVS, Available online: http://www.hgvs.org/mutnomen (accessed on 10 June 2021).

#### 2.4.2. Next Generation Sequencing (NGS)

Genomic DNA extracted from this individual’s sample was used for library preparation and target capture using a custom panel targeting candidate genes. Massively parallel sequencing was performed on the NextSeq 550Dx system (Illumina). Quality control and sequence analysis was performed using our custom analysis pipeline. Copy number analysis was performed using our custom analysis pipeline. GRCh37 (hg19) was used as the reference sequence for mapping and variant calling. Databases used for analysis and variant annotation include Online Mendelian Inheritance in Man (OMIM), Human Gene Mutation Database (HGMD), ClinVar, dbSNP, 1000 Genome, Exome Aggregation Consortium (ExAC), Exome Sequencing Project (ESP), and Korean Reference Genome Database (KRGDB). Classification of variants followed the standards and guidelines established by the American College of Medical Genetics (ACMG). All pathogenic and likely pathogenic variants were further confirmed by Sanger sequencing. This endocrine NGS panel includes 400 genes, including RET, NF1, VHL, SDHA, SDHAF2, SDHB, SDHC, SDHD, FH, MAX, and TMEM127 genes.

### 2.5. Statistical Anlysis

Statistical analysis was performed using SPSS v26.0. χ2 and Fisher exact tests were used for categorical variables. Student’s *t* and Mann–Whitney U tests were applied for continuous variables. Data were considered statistically significant if *p* ≤ 0.05.

The study protocol was approved by the institutional review board (IRB) of the Yonsei University College of Medicine (IRB No.4-2019-1089). The research was performed in accordance with the Declaration of Helsinki. Informed consent was obtained from all patients.

## 3. Results

There were 45 adrenal pheochromocytomas and 7 extra-adrenal but intra-abdominal paragangliomas. All paragangliomas were located in the aortocaval area, which is known as the organ of Zuckerkandl. There were 45 tumours with PASS < 4 and 7 with PASS ≥ 4.

All SDHx mutations with ACMG class 4 or 5 (*n* = 11) were found by NGS. Nine among nine RET mutations, two among four VHL mutations, and two among two NF1 mutations were confirmed by direct sequencing. However, there were four negative results of direct sequencing, even though patients had a family history of MEN2 or past history of hyperparathyroidism. All of these four tumours showed positive SDHB IHC.

The results are presented in Table 1. All the SDHB-negative stained patients had the *SDHB* or *SDHD* mutation. Six of the *SDHB* mutated tumours were pheochromocytomas of the adrenal gland scaled score (PASS) ≥ 4. There was no SDHC mutation found in our cohort. Two of the *RET* and one of the *VHL* mutation cases showed weak diffuse cytoplasmic staining. All 12 remaining tumours were positive for SDHB staining. Of the remaining 26 patients with no pathogenic mutation in gene studies, three were weakly positive for SDHB. Except for the weak positive staining category, the sensitivity and specificity of SDHB IHC for predicting *SDHx* mutations were both 100%.

We performed additional NGS for six patients with weak positive SDHB staining after we confirmed IHC. All six of them showed no pathogenic germline mutation. These two cohorts were combined thereafter, and a total of 58 samples from patients with pheochromocytoma or paraganglioma were stained for SDHB (Table 2). In our study, the SDHB IHC negative group showed a significantly high incidence of high-risk PPGL, with PASS ≥ 4 (*p* = 0.000).

## 4. Discussion

In our first study in South Korea, the results of IHC for SDHB were consistent with those of previous studies. Ten patients with SDHB-negative IHC had *SDHx* mutations. Meanwhile, there were three patients who showed weak positive staining and who had *RET* or *VHL* mutations. These intermediate patterns that associated with other germline mutations rather than *SDH* mutations have been reported by other groups as well [17,22,23]. Previous studies assumed that this might be due to the low concentration of SDHB antibody in the IHC protocol, and it might be correlated with *SDHD* and *VHL* mutations [17,23]. However, in our study, patients with *RET* mutation and no pathogenic germline mutations also showed weak positive staining. This may be explained by false negative results of genetic screening or unknown germline mutations. There was no *SDHx* mutation found in the SDHB-positive group.

*SDHB* mutation is known to correlate with metastatic disease, which is supported by our results. Blank et al. demonstrated that the loss of SDHB can predict metastasis in PPGL, but there was a lack of association with classic hypoxia signalling, which is known to cause malignant PPGL [24]. Although the mechanism of developing malignant PPGL has not yet been identified, the authors suggested more rigorous follow-up of SDHB-negative tumours due to correlation between the loss of SDHB and adverse outcomes [24].

The detection of hereditary PPGL is important for patients as well as their family members, as it increases the risk of developing various multiple and metastatic neoplasms [6]. Mutation analysis for *SDH* genes combined with *RET* and *VHL* gene tests is known to cost up to USD 4100 in the US [25]. Although this might decrease with the adoption of an NGS panel, balancing between risk reduction and financial cost is still an important issue. In this respect, several attempts have been made to develop tools for triaging genetic screening. Meticulous history taking about correlated conditions in syndromic diseases with physical examination, including the typical cutaneous café-au-lait spot in neurofibromatosis type 1, and drawing the familial pedigree can help guide subsequent genetic testing. However, there are no reliable phenotypic differences between sporadic PPGL and hereditary PPGL, which is non-syndromic in nature and caused by germline mutations of *SDHB*, *SDHC*, and *SDHD* genes. For this reason, the WHO recommends genetic testing for all patients even without family history, depending on local resources [21]. A targeted NGS panel has been suggested as a feasible alternative to previous whole exome sequencing for genetic analysis due to its cost-effective nature and easy instrumentation [26]. However, finding an effective way to detect *SDHx* mutations which can help pre-screening will greatly contribute to a further reduction in medical costs and treating hereditary PPGL, especially in developing countries.

SDHA, SDHB, SDHC, and SDHD are encoded by *SDHA*, *SDHB*, *SDHC*, and *SDHD* genes, respectively, and these proteins are assembled in the mitochondria to form the mitochondrial complex II (succinate dehydrogenase), which acts as a key respiratory enzyme as well as an intracellular oxygen sensor [27,28]. It has been demonstrated that the inactivation of any of the *SDH* genes causes functional loss of SDH enzymatic activity, destabilisation, and proteolysis of complex II [17,28]. Thus, the presence of biallelic inactivation of SDH decreases SDHB expression, which can be identified with simple and cheap immunohistochemical staining. The usefulness of IHC staining of SDHB before genetic testing has been validated by many groups. IHC can detect germline *SDHx* mutations with high sensitivity and specificity [8,17]. A three-tiered grading system reported by Gill et al. contributed to increasing the concordance between interobservers [17]. This was further validated by Papathomas et al. with a multinational study [20]. The results of our study also support these previous studies.

However, there are still debates on the interpretation of weakly positive SDHB staining. Menara et al. suggested that further SDHD IHC can help diagnose *SDHx* mutations in the case of weak diffuse SDHB IHC [19]. There were a total of 13 weakly positive SDHB IHC cases in our cohort. Except three patients who had *RET* or *VHL* mutations confirmed by direct sequencing and one patient with *SDHB* mutation confirmed by NGS, the rest of the nine patients who underwent NGS had only ACMG class 3, a variant of unknown significance. According to our study, the possibility of other germline mutations such as *RET* and *VHL* (23%) or negative (or not-yet-known) germline mutations (69%) is higher than that of *SDHx* mutations (8%) when SDHB IHC is weakly positive. Therefore, an additional genetic study would be more helpful than SDHD IHC in the case of weakly positive SDHB IHC.

This study has several limitations. First, it is a retrospective study with small sample numbers. As PPGLs are rare tumours and only those patients who underwent endocrine surgery in the department were included, only 230 patients were screened. As referrals in our centre have been dependent on the location of PPGL, several surgical departments, such as urological surgery, hepatobiliary surgery, neurosurgery, and endocrine surgery, have been involved in the treatment of PPGL. This explains the zero incidence of carotid body paraganglioma and bladder paraganglioma in our study, which has been presented in another study [17]; this could have led to selection bias.

Second, not all the screened patients had undergone genetic analysis. After the exclusion of the genetically untested patients, only 58 patients were enrolled. The cost of an NGS panel for endocrine disease in our centre is approximately USD 4000. Before NGS was introduced, direct sequencing had been used for genetic analysis and it costs about USD 200 per gene. However, not all genes recommended for testing in a recent consensus were available for analysis, such as *FH, MAX*, and *TMEM127* [29]. Although this is cheaper than the cost in the US, the cost is still high and it is a barrier for gene study, especially in the case of patients without medical insurance coverage. Many patients are reluctant to participate in genetic studies because of the sporadic and benign nature of their tumours. The adoption of a target NGS panel specified for PPGL could be helpful to encourage more patients to undergo genetic analysis.

Third, the heterogeneity of the gene study method can also be considered a limitation of this study. As most of the direct sequencing has been performed on single genes according to the family history and correlated symptoms of patients, another untested germline mutation could be neglected. Further large multicentre studies with homogenous multigene screening methods are needed.

Fourth, there is an absence of comparison data with tissue or healthy control samples to confirm germline mutations. Although all mutation analyses have been performed with peripheral blood, this fact alone does not guarantee that all genetic mutations found are germline mutations. However, according to a previous study, genetic mutations in PPGL were almost always associated with germline mutations [17]. Therefore, it can be expected that the mutations found in our study are highly likely germline mutations.

## 5. Conclusions

Despite the above limitations, this is the first study to examine SDHB IHC in PPGL tissues in South Korea, which can add information about the interpretation of weakly positive staining of SDHB. Although immunohistochemistry alone cannot replace genetic evaluation, SDHB IHC can be a reliable and cost-effective preliminary method to screen SDHx mutations. Especially in the case of weakly positive staining of SDHB, an additional study should be considered to rule out *SDHx* or another germline mutation.

## Figures and Tables

**Table 1:** Clinical and immunohistochemical data on 52 patients who underwent genetic analysis.

Gene Mutated	(*n*)	M/F	Age (y, Mean)	PCC (PASS ≥ 4)	PGL (PASS ≥ 4)	SDHB IHC Neg/Weak/Pos
*SDHB*	9	5/4	16–67 (37)	5 (4)	4 (2)	8/1/0
*SDHD*	2	0/2	24–56 (40)	1 (0)	1 (0)	2/0/0
*RET*	9	4/5	13–62 (48)	9 (0)	0	0/2/7
*VHL*	4	3/1	30–55 (48)	3 (0)	1 (0)	0/1/3
*NF1*	2	1/1	36–55 (52)	2 (0)	0	0/0/2
Unknown	26	10/16	19–68 (49)	25 (1)	1 (0)	0/3/23
Total	52	23/29	13–68 (49)	45 (5)	7 (2)	10/7/35

PCC, pheochromocytoma; PGL, paraganglioma; PASS, pheochromocytoma of the adrenal gland scaled score; neg, negative; weak, weakly positive; pos, positive.

**Table 2:** Summary of 58 patients with pheochromocytoma–paraganglioma.

SDHB IHC	PASS	(*n*)	Gene Mutation
SDHB neg	PASS < 4	5	*SDHB* (3), *SDHD* (2)
	PASS ≥ 4	5	*SDHB* (5)
SDHB weak	PASS < 4	12	*RET* (2), *VHL* (1), unknown (9)
	PASS ≥ 4	1	*SDHB* (1)
SDHB pos	PASS < 4	34	*RET* (7), *VHL* (3), *NF1* (2), unknown (22)
	PASS ≥ 4	1	Unknown (1)
Total		58	*SDHB* (9), *SDHD* (2), *RET* (9), *VHL* (4), *NF1* (2), unknown (32)

Neg, negative; weak, weakly positive; pos, positive; PASS, pheochromocytoma of the adrenal gland scaled score.

## Data Availability

Not applicable.
